# Is the Masked Priming Same-Different Task a Pure Measure of Prelexical Processing?

**DOI:** 10.1371/journal.pone.0072888

**Published:** 2013-09-18

**Authors:** Andrew N. Kelly, Walter J. B. van Heuven, Nicola J. Pitchford, Timothy Ledgeway

**Affiliations:** School of Psychology, University of Nottingham, Nottingham, United Kingdom; Stony Brook University, United States of America

## Abstract

To study prelexical processes involved in visual word recognition a task is needed that only operates at the level of abstract letter identities. The masked priming same-different task has been purported to do this, as the same pattern of priming is shown for words and nonwords. However, studies using this task have consistently found a processing advantage for words over nonwords, indicating a lexicality effect. We investigated the locus of this word advantage. Experiment 1 used conventional visually-presented reference stimuli to test previous accounts of the lexicality effect. Results rule out the use of different strategies, or strength of representations, for words and nonwords. No interaction was shown between prime type and word type, but a consistent word advantage was found. Experiment 2 used novel auditorally-presented reference stimuli to restrict nonword matching to the sublexical level. This abolished scrambled priming for nonwords, but not words. Overall this suggests the processing advantage for words over nonwords results from activation of whole-word, lexical representations. Furthermore, the number of shared open-bigrams between primes and targets could account for scrambled priming effects. These results have important implications for models of orthographic processing and studies that have used this task to investigate prelexical processes.

## Introduction

In recent years there has been an increased interest in the early orthographic processes involved in visual word recognition, such as the coding of letter positions in words. This interest has lead to the development of several competing models with various letter position coding schemes [1-9]. One of the problems with testing these models is counterintuitive, as it is not their inability to account for the current experimental data but rather their success at doing so. This means that it is becoming increasingly difficult to differentiate between them on the basis of prevailing evidence. Therefore, new experimental paradigms are needed that can focus on areas that have been previously difficult to investigate and thus overlooked.

For example, there has been surprisingly little research focusing on developing an understanding of the processes involved in letter identification prior to visual word recognition. The neglect of these lower-level processes means that most models of word recognition start after letter identification has been completed, at the "visual word form" level [10]. This means that processes involved in letter perception that may influence later word recognition processes are either left out of models, such as lateral inhibition at the abstract letter level [11], or are assumed to result from later processes.

One reason for this is that the task predominately used for investigating sublexical processes in visual word recognition is the masked priming lexical decision task (for a review, see [12]). The procedure of this task, based on the Forster and Davis [13] paradigm, involves the presentation of a forward mask (e.g., a series of hash marks) which is replaced by the prime letter string, presented very briefly (up to 60 ms) in lowercase font, which is followed immediately by the target letter string in uppercase font. The change in case between the prime and target is generally assumed to make the target act as a backward mask. Participants have to decide whether the target letter string is a correct word or not. The presence of the mask(s) and the brief nature of the prime presentation means that the prime is virtually invisible, hence, the processing of the prime is assumed to be automatic [14]. By manipulating the relationship between the prime and the target (e.g., orthographic similarity) different patterns of priming emerge from which conclusions can be drawn about the representations and processes involved in visual word recognition.

Several different competing theories and models have been developed to explain the effects of the prime on target processing in the lexical decision task (e.g., [13,15,16]). However, it is clear that in order to identify whether or not a letter string is a word a decision is required based on the activation of lexical representations, whether this results from activation of a single lexical representation, multiple lexical representations, or a measure of global lexical activation (e.g., [13,15-17]). Thus, the effects of both lexical processes and stored lexical information can modulate priming effects in the masked priming lexical decision task, limiting its utility for investigating prelexical processes involved in visual word recognition.

As an alternative, the masked priming same-different task has recently been presented as a task that is not affected by higher-level information, such as whole word lexical or phonological information [18,19]. The presentation sequence in the masked priming same-different task is similar to that of the masked priming lexical decision task, however, it differs by the addition of a reference stimulus in lowercase presented above the forward mask, which is clearly visible for one second before it disappears at the same time as the mask. Importantly, the decision in the same-different task is not based on the lexical status of the target (i.e. whether the target is a word or not) as in the lexical decision task, but only on the similarity of the target to the reference (i.e. same or different). Robust orthographic priming for both words and nonwords have been found with the masked priming same-different task (e.g., [18,19]) in contrast to only orthographic priming for words in the lexical decision task (e.g., [18]; also see [12]).

Orthographic priming effects for both words and nonwords in the masked priming same-different task has been used as evidence that the task is not influenced by lexical or phonological information [19]. However, if this task is genuinely free of lexical influences response times should be similar for reference-target pairs that are words or nonwords. Yet results from all versions of the same-different task (unprimed or primed) have showed a consistent advantage for the processing of words (and familiar acronyms) over nonwords (e.g., [19-23]) – an effect that clearly needs to be explained.

Several different accounts have been put forward to explain the word advantage seen in unprimed versions of the same-different task. For example, Chambers and Foster [20] accounted for the word advantage in a three level matching model in which matching can occur at the whole word (lexical), letter cluster, and/or letter level, depending on the nature of the stimuli presented. The model is based on their findings that along with an overall matching advantage for words over nonwords, further advantages occurred for high- over low-frequency words, and legal over illegal nonwords. This, they argued, showed that words were matched at all three levels, with lexical access facilitating the frequency effect along with the overall word advantage. As legal nonwords have no stored lexical representations but contain legal letter clusters they can utilise both the letter cluster and letter levels, but illegal nonwords can only be matched at the letter level. This is consistent with models of word recognition that suggest the encoding of words follows a letter-bigram-word structure (e.g., [4,5,8]).

Marmurek [21] also suggested that lexical units, which are only available for words, are responsible for the word advantage observed in the unprimed version of the same-different task. In addition, he demonstrated that the word advantage is reduced when the reference and target are presented sequentially (as in the masked priming version of the same-different task) compared to simultaneous presentations (as used by Chambers and Forster [20] in the unprimed version of the task). Marmurek proposed that this decrease in the word advantage is due to the creation of new cognitive units for the nonword reference stimuli that are required to successfully complete the task (i.e. some form of temporary memory representation for nonwords is created). Furthermore, Marmurek suggested that the size of the word advantage is dependent on the probability of successfully establishing these memory representations for the nonword stimuli. The implication is that as the strength of the new nonword representation increases it reduces or eliminates the word advantage.

In contrast, Angiolillo-Bent and Rips [24] argued against the hypothesis that words utilise lexical units in the same-different matching task. They investigated the effects of letter displacement in memory encoding by using familiar trigrams (abbreviations such as GDP) and unfamiliar trigrams (e.g., RVT). Participants were required to identify whether the first trigram consisted of the same letters, regardless of position, as a second trigram presented between 500 ms and 2,500 ms later. Despite finding an advantage for processing familiar compared to unfamiliar trigrams this did not interact with the effects of letter displacement or inter-stimulus-interval (ISI) duration. They argued that the lack of interaction indicates that the representations used in the matching process are the same for both familiar and unfamiliar items.

The masked priming same-different task uses sequential presentation of the reference and target. Based on evidence from Marmurek [21] and Angiolillo-Bent and Rips [24] and their own studies, Kinoshita and Norris [19] argued that in this version of the task the representations used for processing the reference would be the same for words and nonwords. Furthermore, they found no interaction between string type and prime type, in Experiment 4 of their study, illustrating that the pattern of priming is similar for words and nonwords. Thus, they posited that the matching process is based on abstract letter representations that are not affected by lexical and/or phonological representations. In this particular experiment (Experiment 4) Kinoshita and Norris manipulated relative letter position across 5 different prime types, (identity, e.g., faith – FAITH; transposed letters (TL), e.g., fiath – FAITH; two substituted letters (2L Sub), e.g., fouth – FAITH; scrambled, e.g., ifhat – FAITH; and unrelated, e.g., agent - FAITH). Despite finding no significant interaction between string and prime type, a significant advantage for the processing of words over nonwords was found. Kinoshita and Norris argued that the advantage for processing words over nonwords reflects differences in the ease of processing familiar items.

Kinoshita [25] explained familiarity as a global measure that operates before or during the processes involved in encoding/identifying individual letters. To date studies using the masked priming same-different task have suggested that the performance effects that arise within this task are based on representations occurring at or after the abstract letter level because the same pattern of priming is found for both words and nonwords (e.g., [18,19,26]). This finding also rules out the possibility that low-level perceptual processes contribute to the word advantage in this task, as any perceptual effect would occur before the abstract letter level and therefore would apply to both words and nonwords. Importantly, in the masked priming version of the same-different task, factors that influence lexical access, such as frequency and neighbourhood density, have been shown not to modulate performance ([18,27], respectively). Although this suggests that higher-level lexical information does not influence the processing of the prime and target, it does not preclude sublexical orthographic influences (e.g., bigrams).

Recently, Kinoshita and Lagoutaris [28] argued that orthotactic knowledge is used for encoding the reference in the masked priming same-different task. They proposed that the representation of the reference is held in visual short-term memory (similar to the "graphemic buffer" first proposed for spelling e.g., [29]). Orthotactic knowledge is used to either reconstruct or reintegrate decaying memory traces and thus allowing orthographically legal, pronounceable, nonwords containing more than four letters to be successfully stored in visual short-term memory (which is presumed to have a capacity equal to or less than four). Kinoshita and Lagouyaris described this orthotactic knowledge as being at a higher level than that of abstract letter representations, however no further specification was given.

A second possibility is that different orthotactic information is used for encoding word and nonword reference stimuli. As discussed earlier, Chambers and Forster [20] suggested that matching of the reference and target could occur at three different levels depending on the nature of the letter string, with words matching at the letter, letter cluster, and word level, and pronounceable nonwords matching at the letter and cluster levels. Thus, the word advantage could result from the utilisation of different sized units when encoding and supporting the representation of the reference stimuli, with words being encoded as a single unit supported by their lexical representations and nonwords being encoded as orthotactic chunks. These "chunks" could be phonologically-based graphemes or purely orthographically-based letter combinations, such as bigrams, which could be contiguous bigrams (e.g., BL in BLANK), noncontiguous open-bigrams (e.g., BA in BLANK), or larger units, such as rhymes (e.g., OUGH, IGHT).

**Figure 1:**
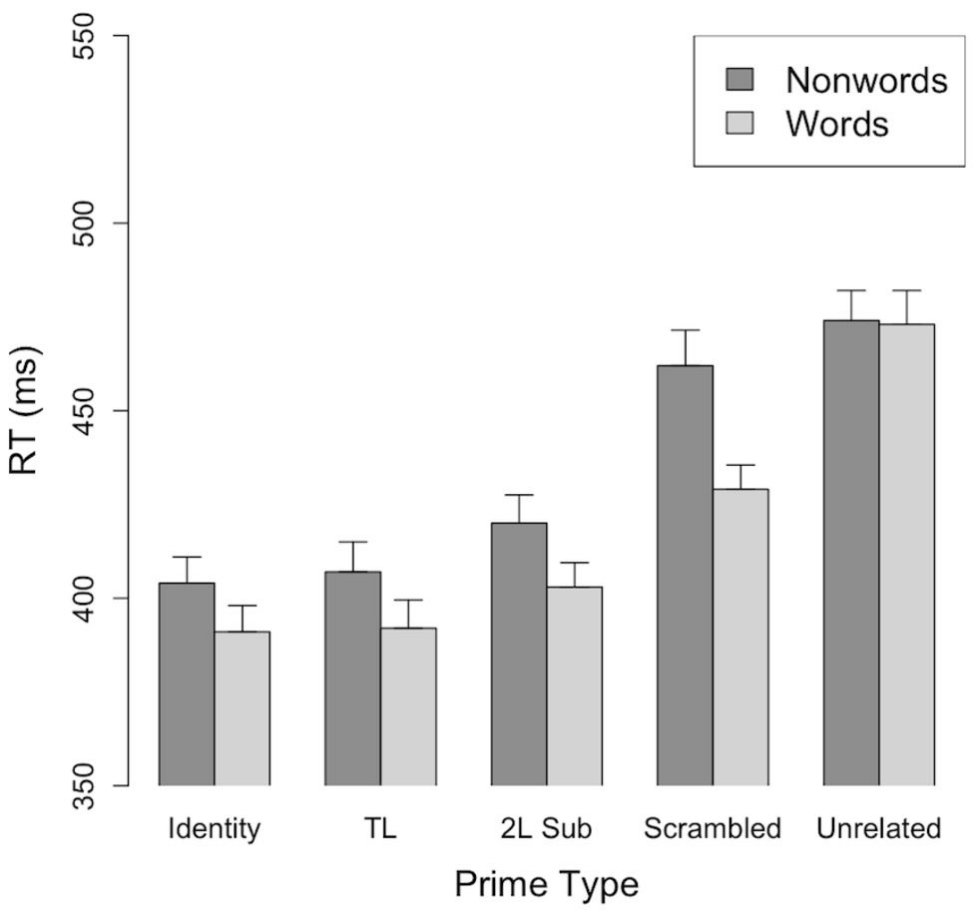
Mean response times for Experiment 4 of Kinoshita and Norris [19]. Error bars indicate the standard error of the mean.

Whatever the nature of orthotactic knowledge, it is important to note that the lack of interaction between prime type and string type in the studies of Angiolillo-Bent and Rips [24] and Kinoshita and Norris [19] indicates that lexical processes do not modulate performance in the masked priming same-different task. However, close inspection of the mean response times of Experiment 4 in Kinoshita and Norris [19] suggests the possibility of an interaction between two of the five priming conditions (scrambled, e.g., ifhat - FAITH and unrelated, e.g., agent - FAITH). As illustrated in Figure 1, there appears to be no word advantage for unrelated primes and no scrambled priming effect for nonwords.

The three critical priming conditions in Kinoshita and Norris [19] are identity, scrambled, and unrelated. These priming conditions provide critical comparisons, as the only difference between identity and scrambled primes is the absence of correct positional information in the latter condition. Thus, identity primes share both letter identity and positional information with the target, whereas scrambled primes share only letter identity information with the target. The difference between scrambled and unrelated primes arises from access to letter identity information in the scrambled, but not in the unrelated prime condition. Thus, scrambled and identity primes can produce priming at different levels of processing: identity priming at the letter, letter cluster (e.g., grapheme, bigram) and lexical (word) level and scrambled priming at the letter level only. An interaction between these three primes and string type indicates that matching in the same-different task occurs at different levels, whereas no interaction rules out matching at multiple levels. To test this hypothesis, we conducted an experiment (Experiment 1a) with just these three prime conditions of Kinoshita and Norris [19].

In the masked priming same-different task the reference stimulus is presented for one second – sufficient time for "one-trial" learning which could support long-term priming (see [30,31]). This should enable successful encoding of the reference for immediate use in the matching process. Varying the presentation time of the reference stimulus should thus affect the extent of the advantage shown for words over nonwords in the masked priming same-different task. Marmurek [21] suggested that extending the ISI should reduce the size of the word advantage, as more time is available to encode the nonword reference stimulus. This hypothesis was tested in Experiment 1b by extending the presentation time of the reference stimulus from one second (as in Experiment 1a) to two seconds. We further tested this hypothesis in Experiment 1c by reducing the presentation time of the reference stimuli to 500 ms. This reduction in presentation time should increase the size of the word advantage, as less time is available to encode the nonword reference stimulus in visual short-term memory.

An alternative explanation for the word advantage shown in the masked priming same-different task is that different processing strategies are used for word and nonword stimuli, based on the predictability of the target string type. In the standard task procedure, reference-target pairs consist of the same string type (either both words or nonwords) even in the different condition, when the reference stimulus differs from the target (e.g., often – DRUMS). Thus, target string type is highly predictable from the reference stimulus within any one trial. If the reference stimulus is a word this may induce lexical strategies, whereas if the reference stimulus is a nonword, sublexical strategies may be employed. Several studies using repetition proportion (RP) priming have demonstrated that masked priming is susceptible to the use of strategies (e.g., [32-34]). RP priming effects occur when the proportion of experimental primes appearing in the task, compared to control primes, are manipulated, with higher proportions of experimental primes generally resulting in larger priming effects (e.g., [32-34]). These RP priming effects have been argued to demonstrate that the cognitive system automatically changes the level of influence the prime has on processing the target depending on the probability that the prime will be of use in the task [35]. Although the proportion of primes are not different in the masked priming same-different task, the design involves blocks of target strings of the same type, therefore the target string type is highly predictable *between*-trials. We tested the hypothesis that strategic effects in processing the reference stimulus result in a word advantage on the masked priming same-different task in Experiment 1d by removing the blocking of trials by string type to reduce between-trials predictability, and also by mixing string type across reference-target pairs in the different condition to reduce predictability *within*-trials.

## Experiment 1a

### Methods

#### Ethics statement

This experiment was approved by the School of Psychology Ethics Committee, University of Nottingham. Written informed consent was obtained from all participants prior to participation.

#### Participants

Twenty-four students (17 females and 7 males with an average age of 21.4 years) took part in this experiment. All were native English speakers with normal or corrected-to-normal vision.

#### Stimuli and design

Critical stimuli for the "same" trials were taken from Kinoshita and Norris [19]. These consisted of 78 five-letter words, 78 nonwords, and three groups of 78 primes (identity, scrambled and unrelated). The identity prime was the same as the target (e.g., faith-FAITH). The scrambled prime was a 31524 permutation for five-letter strings when denoted as 12345, ensuring none of the letters: 1) appeared in the same position, 2) were adjacent to the same letters that they were adjacent to in the original string (i.e. no transposition of adjacent letters), and 3) relative positioning was removed, for example, ifhat-FAITH. For the unrelated primes 26 five-letter words were used, 20 from the Kinoshita and Norris study and due to the reduction in the number of priming conditions increasing the number of trials per prime from 20 to 26 an additional six words were needed which were matched in characteristics to the original 20.

As the non-critical stimuli, those used for the "different" trials, from the Kinoshita and Norris [19] study were not available, 156 five-letter filler words (78 used as target stimuli and 78 as reference stimuli) were selected using the same criteria as the original study. The words were matched in characteristics to the critical condition words and the three priming conditions were constructed using the same methods as for the critical target words. Each target word was paired with one reference word, for example, reference: anger, target: MONTH. To produce the 156 filler nonwords and their corresponding primes one letter was changed in each filler word.

The design was identical to that used by Kinoshita and Norris [19]. It involved a counterbalanced blocked presentation of words and nonwords. Each of the four groups of target stimuli ("same" and "different" trials words and nonwords) were separated into three groups and assigned different prime conditions across three lists. This allowed each target item to be presented to each participant once only but in a different priming condition. Thus six lists were used and each list consisted of 156 target words (78 critical and 78 filler) and 156 target nonwords (78 critical and 78 filler), 78 identity, scrambled and unrelated primes; 26 of each for the four groups of target stimuli. Each participant was randomly assigned to one of the six lists.

#### Procedure

The procedure was identical to Kinoshita and Norris [19]. Each trial started with a forward pattern mask consisting of five hash marks (# # # # #) presented in the centre of the screen and the reference stimulus in lower case directly above, which remained on the screen for 1000 ms. This was followed by the prime in lower case, which was presented for 37 ms, then the target stimulus was presented in upper case and remained on the screen until either a response was made or 2000 ms had passed. After each trial a blank screen was presented for 500 ms before the next trial started. DMDX [36] was used to present the stimuli and record the responses. All responses were made using an external button box connected to the computer. Each participant was tested separately. The stimuli were high contrast and presented in a white Courier New font (10 point) on a black background. The participants were instructed to attend to the letter string presented above the string of hash marks. When these disappeared a second letter string would replace the hash marks. The participants were then asked to decide as quickly and accurately as possible whether the new letter string presented in upper case was the same or different than the first letter string, ignoring the change in case, by pressing the right button if it was the same and the left button if it was different. The presence of a prime was not mentioned. Each participant completed 328 trials in total, comprising sixteen practice and 312 test trials. All trials within each block were presented in a randomized order. Response times were measured in milliseconds from the onset of the target stimulus.

### Results

Analyses were run on both the mean correct response times (RT) and the percentage of errors (total 4.2%). Trials with latencies above 1400 ms and below 250 ms were excluded from the analyses (0.2% of the trials). The "same" and "different" trials were analysed separately using a two-way repeated measures ANOVA with String Type (words or nonwords) and Prime Type (identity, scrambled or unrelated) using both by-participant (F_1_) and by-item (F_2_) analyses. These analyses were conducted in this and subsequent experiments. Mean RTs to correct trials and error rates are presented in Table 1.

**Table 1:** Mean response times in milliseconds, percentage error rate, and standard error (SE) of the means of Experiment 1a.

Trials	String Type	Prime Type	Prime - Target Pair Examples	Response Times (SE)	% Error (SE)
"Same"	Words	Identity	flair - FLAIR ^a^	419 (14)	3.5 (0.7)
		Scrambled	afrli - FLAIR ^a^	449 (16)	4.5 (0.9)
		Unrelated	panel - FLAIR ^a^	465 (13)	7.5 (1.3)
	Nonwords	Identity	ditle - DITLE ^b^	447 (16)	3.4 (0.9)
		Scrambled	tdeil - DITLE ^b^	469 (16)	5.4 (1.2)
		Unrelated	glimb - DITLE ^b^	491 (14)	7.9 (1.2)
"Different"	Words	Identity	drums - DRUMS ^c^	483 (14)	3.8 (0.5)
		Scrambled	udsrm - DRUMS ^c^	489 (13)	4.1 (0.8)
		Unrelated	acted - DRUMS ^c^	489 (14)	5.1 (0.8)
	Nonwords	Identity	benor - BENOR ^d^	496 (18)	3.7 (0.7)
		Scrambled	nbroe - BENOR ^d^	493 (16)	4.0 (0.7)
		Unrelated	acide - BENOR ^d^	497 (15)	5.8 (0.8)

a Reference: flair

b Reference: ditle

c Reference: often

d Reference: ampty

#### "Same" trials

For the response latencies the main effect of String Type was significant, *F*_1_(1, 23) = 12.60, *p* < .01, *F*_2_(1, 155) = 41.06, *p* < .001 with responses to words 25 ms faster than those to nonwords, indicating a processing advantage for words. The main effect of Prime Type was also significant, *F*_1_(2, 22) = 65.84, *p* < .001, *F*_2_(2, 154) = 41.71, *p* < .001. There was no interaction between String Type and Prime Type, all *F*s < 1, therefore RTs were collapsed across String Type. Subsequent planned comparisons revealed relative to the unrelated condition facilitation effects for the identity, *F*_1_(1, 23) = 152.15, *p* < .001, *F*_2_(1, 155) = 80.13, *p* < .001, and scrambled conditions *F*_1_(1, 23) = 20.14, *p* < .001, *F*_2_(1, 155) = 13.59, *p* < .001. Furthermore, the identity condition differed significantly from the scrambled condition, *F*_1_(1, 23) = 44.65, *p* < .001, *F*_2_(1, 155) = 24.37, *p* < .001. The mean RTs for the identity primes were 26 ms faster than the scrambled primes, which were 19 ms faster than unrelated primes.

No significant main effect of String Type was found in the error rates, all *F*s < 1. There was a main effect of Prime Type, *F*_1_(2, 46) = 7.25, *p* < .01, *F*_2_(1, 153) = 11.15, *p* < .001. There was no interaction between the variables, all *F*s < 1. Planned comparison carried out on the error rates collapsed across String Type showed, as for the RTs, significant priming effects for the identity and scrambled conditions, *F*_1_(1, 23) = 11.71, *p* < .01, *F*_2_(1, 155) = 20.46, *p* < .001, and *F*_1_(1, 23) = 6.52, *p* < .05, *F*_2_(1, 155) = 7.65, *p* < .01 respectively. There was no significant difference between the identity and scrambled condition F_1_ < 1, *F*_2_(1, 155) = 3.69, *p* = .06. Thus, identity and scrambled primes were responded to more accurately then unrelated primes (3.5% and 5% versus 7.7%).

#### "Different" trials

For the RTs there were no significant effects for String Type, *F*_1_(1, 23) = 1.05, p = .32, *F*_2_(1, 155) = 2.98, *p* = .09, Prime Type, or interaction, all *F*s < 1. In the error rates no main effect for String Type was found, *F*s < 1, but there was a significant main effect of Prime Type *F*_1_(2, 22) = 4.28, *p* = .02, *F*_2_(2, 154) = 11.15, *p* < .001. There was no interaction, *F*s < 1. Collapsed across String Type error rates revealed significantly less errors for both the identity and the scrambled conditions relative to the unrelated condition, *F*_1_(1, 23) = 7.96, *p* < .01, *F*_2_(1, 155) = 20.46, *p* < .001, and *F*_1_(1, 23) = 4.73, *p* < .05, *F*_2_(1, 155) = 7.65, p < .01. There was no significant difference between the identity and scrambled prime conditions, *F*s < 1.

### Discussion

The results from Experiment 1a revealed that, for same responses, times to words were faster than those to nonwords. Furthermore, significant priming effects for both the identity and scrambled primes were found, with identity primes producing larger facilitation effects than the scrambled primes. Critically there was no interaction between string type and prime type, consistent with Kinoshita and Norris [19]. However, our results differ from those of Kinoshita and Norris in two key findings. First, we found clear numerical differences between the response times of the words and nonwords in the unrelated priming condition, and second the priming effect of the scrambled condition was similar in size for both words and nonwords (22 ms and 16 ms respectively as opposed to 44 ms and 12 ms in Kinoshita and Norris [19] Experiment 4). As noted previously, it was the apparent lack of these two effects in Kinoshita and Norris’ experiment that led us to suspect that an interaction might exist between string and prime type if only the three critical primes conditions employed here were used. However, we also found the word advantage did not interact with prime type. Nonetheless, the advantage shown for processing words over nonwords in this experiment, and other studies using the same-different task, still requires explanation. We systematically test the accounts given in the introduction in Experiments 1b-d.

## Experiments 1b and 1c

The aim of Experiments 1b and 1c was to test the prediction that the word advantage results from a difference in the strength of representation of the reference stimulus. It is possible to modulate the strength of a nonword reference representation by changing the reference presentation time. Extending the duration of the reference stimulus should increase the strength of representation for nonwords, which should in turn reduce the size of the word advantage [21]. Likewise, reducing the duration of the reference stimulus should reduce the strength of representation for nonwords, which in turn should increase the size of the word advantage. In Experiment 1b the reference duration used in Kinoshita and Norris [19] and in the previous experiment was increased to 2 seconds, and in Experiment 1c it was reduced to 500 ms.

### Methods

#### Ethics statement

The experiments were approved by the School of Psychology Ethics Committee, University of Nottingham. Written informed consent was obtained from all participants prior to participation.

#### Participants

In this experiment a total of seventy-four undergraduate students participated in exchange for course credit, with forty-one in Experiment 1b and thirty-three in Experiment 1c. All were native English speakers with normal or corrected-to-normal vision.

#### Stimuli and design and procedure

**Table 2:** Mean response times in milliseconds, percentage errors, and standard error (SE) of the means of Experiment 1b-d.

			Experiment 1b		Experiment 1c		Experiment 1d	
Trials	String Type	Prime Type	Response Times (SE)	% Error (SE)	Response Times (SE)	% Error (SE)	Response Times (SE)	% Error (SE)
"Same"	Words	Identity	657 (18)	4.0 (0.8)	581 (16)	3.7 (1)	401 (8)	3.2 (0.8)
		Scrambled	688 (18)	4.6 (0.6)	590 (14)	4.0 (0.8)	427 (11)	6.6 (1.0)
		Unrelated	704 (17)	5.5 (0.8)	612 (14)	7.8 (1.4)	462 (9)	9.3 (1.8)
	Nonwords	Identity	690 (24)	5.5 (0.7)	607 (17)	3.8 (0.7)	429 (8)	6.6 (0.8)
		Scrambled	717 (22)	6.7 (1.1)	624 (14)	4.1 (0.8)	455 (11)	7.1 (1.2)
		Unrelated	728 (23)	6.9 (0.9)	631 (15)	5.4 (0.9)	479 (10)	11.7 (2.3)
"Different"	Words	Identity	732 (19)	4.7 (0.7)	655 (17)	2.9 (0.6)	482 (19)	3.1 (0.6)
		Scrambled	733 (18)	4.8 (0.6)	657 (16)	3.1 (0.5)	485 (22)	4.4 (0.9)
		Unrelated	741 (19)	4.5 (0.6)	644 (14)	5.6 (0.7)	481 (19)	5.6 (1.5)
	Nonwords	Identity	741 (22)	5.4 (0.8)	664 (18)	4 (0.5)	482 (20)	3.6 (0.8)
		Scrambled	759 (23)	6.1 (1)	667 (17)	3.6 (0.6)	486 (19)	3.8 (0.9)
		Unrelated	742 (22)	5.7 (0.7)	661 (16)	4.4 (0.5)	493 (21)	5.6 (1.5)

The stimuli, design, and procedure for these two experiments were the same as those described in Experiment 1a, except that in Experiment 1b both the reference and the forward mask were presented for 2000 ms and in Experiment 1c they were presented for 500 ms.

### Results: Experiment 1b

Response latencies above 1400 ms and below 250 ms were excluded from the analyses (1.9% of all trials) to remove outliers. The overall error rate was 3.3%. Mean RTs and error rates are presented in Table 2.

#### "Same" trials

Responses to nonwords were 29 ms slower than to words, *F*_1_(1,40) = 7.6, *p* < .01, *F*_2_(1,155) = 40.61, *p* < .001. A main effect of Prime Type was found, *F*_1_(2,39) = 32.59, *p* < .001, *F*_2_(2,154) = 19.3, *p* < .001 and there was no interaction between String Type and Prime Type, *F*s < 1. Data were therefore collapsed across String Type and planned comparisons were conducted. These revealed a facilitation effect for the identity and scrambled primes relative to the unrelated prime condition, *F*_1_(1,40) = 47.54, *p* < .001, *F*_2_(1,155) = 39.57, *p* < .001 and *F*_1_(1,40) = 7.12, *p* < .01, *F*_2_(1,155) = 4.81, *p* < .05, respectively. Furthermore the identity prime condition differed significantly from the scrambled prime condition, *F*_1_(1,40) = 37.74, *p* < .001, *F*_2_(1,155) = 14.75, *p* < .001. Thus responses for identity primes were faster (28 ms) then those for scrambled primes, which were faster (14 ms) than unrelated primes.

There was a significant main effect in the error rate for String Type, *F*_1_(1,40) = 8.39, *p* < .01, *F*_2_(1,155) = 9.41, *p* < .01, with nonwords producing more errors than words (6.4% versus 4.7%). There was an effect of Prime Type by-participant, *F*_1_(2,39) = 3.05, *p* = .05, but not by-item, F_2_ < 1. However, there was no interaction, *F*s < 1.

#### "Different" trials

A marginal significant effect was found in the RTs for String Type by-participant, *F*_1_(1,40) = 2.95, *p* = .09, and a significant effect by-item, *F*_2_(1,155) = 7.44, *p* < .01. There were no main effect of Prime Type, *F*_1_(2,39) = 2.22, *p* = .23, F_2_ < 1 and a significant interaction by-participant, *F*_1_(2,39) = 3.83, *p* < .05, but not by-item, *F*_2_(2,154) = 1.56, *p* = .21.

The analysis of the error rates revealed a significant main effect of String Type, *F*_1_(1,40) = 4.09, *p* < .05, *F*_2_(1,155) = 7.86, *p* <.01, with more errors made to nonwords than words (5.7% versus 4.6%). There was no significant effect of Prime Type, or interaction, *F*s <1.

### Results: Experiment 1c

Trials with latencies above 1400 ms and below 250 ms were excluded from the analyses (0.4% of the trials). The overall error rate was 3.5%. Mean RTs and errors rates are presented in Table 2.

#### "Same" trials

For the responses latencies the main effect of String Type was significant, *F*_1_(1,32) = 9.56, *p* < .01, *F*_2_(1,155) = 48.368, *p* < .001, with responses to words 27 ms faster than those to nonwords. The main effect of Prime Type was also significant, *F*_1_(2,31) = 9.3, *p* < .001, *F*_2_(2,154) = 25.84, *p* < .001. There was no interaction between String and Prime Type, *F*_1_(2,31) = 1.81, *p* = .17, *F*_2_(2,154) = 1.72, *p* = .18, so RTs were collapsed across String Type. Subsequent planned comparisons revealed relative to the unrelated condition facilitation effects for both the identity and scrambled conditions, *F*_1_(1,32) = 11.23, *p* < .01, *F*_2_(1,155), 55.98, *p* < .001, and *F*_1_(1,32) = 7.03, *p* < .05, *F*_2_(1,155) = 8.24, *p* < .01, respectively. Furthermore, the identity condition differed significantly from the scrambled condition, *F*_1_(1,32) = 6.81, *p* < .05, *F*_2_(1,155) = 17.12, *p* < .001. The mean RTs for identity primes were 13 ms faster than those for scrambled primes, which were 14 ms faster than unrelated primes.

No significant main effect of String Type was found in the error rates, *F*_1_(1,32) = 1.56, *p* = .22, *F*_2_(1,155) = 1.53, *p* =.21. There was a main effect of Prime Type, *F*_1_(2,31) = 5.14, *p* < .01, *F*_2_(2,154) = 4.32, *p* < .05, but there was no interaction, *F*_1_(2,31) = 1.47, *p* = .24, *F*_2_(2,154) = 1.74, *p* = .18. Planned comparisons conducted on error rates collapsed across String Type showed unrelated primes differed significantly from identity primes, *F*_1_(1,32) = 5.53, *p* < .05, *F*_2_(1,155) = 4.94, *p* < .05, and scrambled primes by-participant, *F*_1_(1,32) = 7.72, *p* < .01, but not by-item, F_2_ < 1. There was no difference between identity and scrambled primes, *F*s < 1. Thus, identity and scrambled primes were responded to more accurately than unrelated primes (3.8% and 4% versus 6.6%).

#### "Different" trials

For the RTs the effect of String Type was not significant by-participant, *F*_1_(1,32) = 2.09, *p* = .16, but significant by-item, *F*_2_(1,155) = 8.49, *p* < .01. There was no significant main effect of Prime Type, *F*_1_(2,31) = 2.39, *p* = .10, F_2_ < 1, or interaction, *F*s < 1. In the error rates no main effect of String Type was found, *F*s < 1, but there was a significant main effect of Prime Type by-participant, *F*_1_(2,31) = 6.64, *p* < .01, but not by-item, F_2_ <1. The interaction between these factors was marginal by-participant, *F*_1_(2,31) = 2.7, *p* =.07, and significant by-item, *F*_2_(2,154) = 3.38, *p* < .05.

### Discussion

The results for Experiment 1b and 1c showed the same overall pattern of results as Experiment 1a with faster responses times to words than to nonwords. Critically the size of the word advantage was almost identical for both the long and short reference presentation duration (29 ms vs. 27 ms) and the original presentation duration of the reference stimulus used in Experiment 1a (25 ms). As in Experiment 1a, no significant interaction between string and prime type was found. This demonstrates that the word advantage does not arise from differences in the strength of representations established for the reference stimulus that are used in the matching process.

## Experiment 1d

The aim of Experiment 1d was to see if the advantage for words over nonwords found in the masked priming same-different task arises from different strategies being employed when processing word and nonword reference-target pairs. Thus, in this experiment blocking by stimulus type between-trials was removed to reduce the predictability of the stimuli presented on consecutive trials and lessen the effectiveness of strategy use in this task. In addition, to eliminate within-trial predictability of the target stimulus from the reference stimulus string type, reference-target pairs in the "different" trials were mixed so that the reference string type could no longer be used to predict the string type of the target.

### Methods

#### Ethics statement

This experiment was approved by the School of Psychology Ethics Committee, University of Nottingham. Written informed consent was obtained from all participants prior to participation.

#### Participants

Twenty-four undergraduate and postgraduate students (18 females and 6 males with an average age of 21.1 years) from the University of Nottingham were recruited to this experiment. All were native English speakers with normal or corrected-to-normal vision. None of them had participated in Experiments 1a, 1b or 1c.

#### Stimuli and design

Stimuli were the same as in Experiment 1a. The design was also the same as in Experiment 1a except that for the 156 filler target items (i.e. those requiring a "different" response) half of the 78 target words were paired with nonword reference stimuli and vice versa for nonword targets (e.g., reference: often – target: MUNDS). Blocking of word and nonword trials was also removed hence all trials were presented in a randomized order. Three stimulus lists were constructed which were presented to an equal number of participants.

#### Procedure

The procedure was the same as in Experiment 1a.

### Results

Trials with latencies above 1400 ms and below 250 ms were excluded from the analyses (0.1% of trials). The overall error rate was 5.1%. Mean RTs and error rates are presented in Table 2. An additional variable of Reference-Target Pair (consistent or inconsistent) was included in the analysis of the "different" trials.

#### "Same" trials

Responses to nonwords were 25 ms slower than to words, *F*_1_(1, 23) = 44.07, *p* < .001, *F*_2_(1, 155) = 25.67, *p* < .001. A main effect of Prime Type was found, *F*_1_(2, 22) = 55.53, *p* < .001, *F*_2_(2, 154) = 66.15, *p* < .001 and there was no interaction between Prime Type and String Type, *F*s < 1. Data were collapsed across String Type and planned comparisons were conducted. These revealed a facilitation effect for the identity and scrambled primes relative to the unrelated primes, *F*_1_(1, 155) = 103.35, *p* < .001, *F*_2_(1, 23) = 151.30, *p* < .001 and *F*_1_(1, 155) = 33.82, *p* < .001, *F*_2_(1, 23) = 35.61, *p* < .001 respectively. Furthermore, the identity primes differed significantly from the scrambled primes, *F*_1_(1, 155) = 11.475, *p* < .01, *F*_2_(1, 23) = 24.26, *p* < .001. Thus, identity primes were responded to faster (26 ms) than scrambled primes, which were faster (30 ms) than unrelated primes.

There was a significant main effect in the error rates of String Type, *F*_1_(1, 23) = 4.22, *p* = .05, *F*_2_(1, 153) = 3.94, p < .05, and Prime Type, *F*_1_(2, 46) = 6.32, *p* < .01, *F*_2_(1, 153) = 9.56, *p* < .001, but again, no interaction between these variables was observed, *F*_1_(2, 46) = 1.62, *p* = .21, F_2_ < 1. Planned comparisons revealed significantly less errors in the identity and scrambled prime conditions relative to the unrelated prime condition, *F*_1_(1, 23) = 7.36, *p* < .05, *F*_2_(1, 155) = 16.53, *p* < .001, and *F*_1_(1, 23) = 5.00, *p* < .05, *F*_2_(1, 155) = 6.77, *p* < .01, respectively. Error rates in the identity prime condition were significantly less than in the scrambled prime condition by-participant, *F*_1_(1, 23) = 5.11, *p* < .05, and marginally by-item*, F*_2_(1, 155) = 3.14, *p* = .08.

#### "Different" trials

No significant effects were found in the RTs for String Type, *F*_1_(1, 23) = 1.49, *p* = .24, F_2_ < 1, Reference-Target Pair, *F*_1_(1, 23) = 1.93, *p* = .18, *F*_2_(1, 155) = 1.07, *p* = .30, and Prime Type, and no interaction, all *F*s < 1.

The analyses of the error rates revealed no differences between word and nonword targets, *F*s < 1, but a significant effect of Prime Type, *F*_1_(2, 22) = 5.78, *p* < .01, *F*_2_(2, 154) = 9.85, *p* < .001, and Reference-Target Pair, *F*_1_(1, 23) = 12.80, *p* < .01, *F*_2_(1, 155) = 15.67, *p* < .001 with a lower error rate for inconsistent than for consistent reference-target pairs. There were no interactions between String Type and Prime Type, *F*s < 1, String Type and Reference-Target Pair, *F*_1_(2,154) = 2.15, *p* = 0.15, *F*_2_(2,154) = 1.31, *p* = 0.25, Prime Type and Reference-Target Pair, *F*_1_(2,154) = 1.93, *p* = 0.15, F_2_ < 1, and String Type, Prime Type and Reference-Target Pair, *F*_1_(2,154) = 1.27, *p* = 0.29, F_2_ < 1. Planned comparisons revealed that there were significantly less errors for both the identity and the scrambled prime conditions than the unrelated prime condition, *F*_1_(1, 23) = 8.10, *p* < .01, *F*_2_(1, 155) = 15.87, *p* < .001, and *F*_1_(1, 23) = 4.24, *p* = .05, *F*_2_(1, 155) = 8.43, *p* < .01, respectively. There was no significant difference between the identity and scrambled prime conditions, *F*_1_(1, 23) = 2.67, *p* = .12, *F*_2_(1, 155) = 2.1, *p* = .15.

### Discussion

Results of Experiment 1d mirror those found in Experiments 1a-c. Words were processed faster than nonwords and critically there was again no interaction between string and prime type. These results suggest that the predictability of the target string type did not influence the pattern of priming effects found in the masked priming same-different task. Thus, blocking trials by stimulus type, and pairing reference and target stimuli by string type, did not induce the use of different strategies for processing words and nonwords in this task.

Whilst Kinoshita and Norris [19] argued that the same representations are used in the matching process for words and nonwords this seems unlikely because there is a consistent word advantage, as shown clearly in Experiments 1a-d. Rather, the results of our experiments suggest that the word advantage may arise from differences in the representations involved in matching the reference and target. As suggested by Chambers and Forster [20] matching could occur at several levels depending on the type of string used, with nonwords matching at the sublexical level and words matching at both the sublexical and lexical level. To test which level is used for the matching process we conducted a further experiment in which the presentation modality of the reference was changed from visual to auditory.

When the reference stimulus is presented in the auditory modality the matching process could occur at the phonological level through the target being converted into a phonological code. For words this could occur at the lexical or sublexical level but for nonwords this is only possible sublexically. When letter order is preserved, as in identity primes, conversion of the target to phonology is facilitated for both words and nonwords, but when letter order is disrupted, as in scrambled primes, conversion of the target to phonology is not facilitated at the sublexical level. However, scrambled primes could still potentially facilitate the processing of word targets at the lexical level through activation of shared sublexical orthographic representations (e.g., open-bigrams). In contrast, scrambled priming effects would not occur for nonword targets because they do not have lexical representations.

## Experiment 2a

### Methods

#### Ethics statement

This experiment was approved by the School of Psychology Ethics Committee, University of Nottingham. Written informed consent was obtained from all participants prior to participation.

#### Participants

Twenty-four undergraduate and postgraduate students (19 females and 5 males with an average age of 23.1 years) from the University of Nottingham participated in this experiment. All were native English speakers with normal or corrected-to-normal vision and none had participated in the previous experiments.

#### Stimuli and design

Stimuli were the same as in Experiment 1a. Reference stimuli were recorded using a female adult speaker with a non-specific English accent. Audio was recorded with a sampling rate of 44,100 Hz and edited using Amadeus Pro (www.hairersoft.com/AmadeusPro/). Each of the audio files was edited so that the total duration was 1 second (the same duration that the hash marks remained on the screen), and the offsets of the audio stimulus and hash marks were synchronous. The design used for this experiment was the same as Experiment 1a, because the pattern of results was identical across Experiments 1a-d.

#### Procedure

The procedure was the same as Experiment 1a, except that the reference stimuli were presented in the auditory rather than visual domain.

### Results

Trials with latencies above 1400 ms or below 250 ms were removed from the analyses, accounting for 0.3% of the total data. The overall error rate was 3.6%. The mean RTs and error rate are given in Table 3.

**Table 3:** Mean response times in milliseconds, percentage error rate, and standard error (SE) of the means of Experiment 2a.

Trials	String Type	Prime Type	Prime - Target Pair Examples	Response Times (SE)	% Error (SE)
"Same"	Words	Identity	flair - FLAIR ^a^	447 (14)	2.2 (0.9)
		Scrambled	afrli - FLAIR ^a^	485 (15)	3.5 (0.9)
		Unrelated	panel - FLAIR ^a^	515 (14)	4.8 (1.1)
	Nonwords	Identity	ditle - DITLE ^b^	509 (19)	7.1 (1.6)
		Scrambled	tdeil - DITLE ^b^	539 (15)	7.1 (1.0)
		Unrelated	glimb - DITLE ^b^	543 (15)	9.3 (1.0)
"Different"	Words	Identity	drums - DRUMS ^c^	530 (17)	2.5 (0.6)
		Scrambled	udsrm - DRUMS ^c^	532 (19)	3.2 (0.6)
		Unrelated	acted - DRUMS ^c^	541 (20)	3.5 (0.7)
	Nonwords	Identity	benor - BENOR ^d^	546 (15)	4.5 (0.8)
		Scrambled	nbroe - BENOR ^d^	541 (15)	4.1 (0.5)
		Unrelated	acide - BENOR ^d^	553 (16)	5.5 (0.5)

a Reference: flair

b Reference: ditle

c Reference: often

d Reference: ampty

#### "Same" trials

The analysis of RT latencies for the "same" trials revealed a significant effect of String Type, *F*_1_(1, 23) = 27.74, *p* < .001, *F*_2_(1, 155) = 36.15, *p* < .001, with responses to nonwords 48 ms slower than to words. The effect of Prime Type was also significant, *F*_1_(2, 22) = 31.04, p < .001, *F*_2_(2, 154) = 29.43, p < .001. In contrast to our previous experiments, there was a significant interaction between String Type and Prime Type, *F*_1_(2, 22) = 3.25, *p* < .05, *F*_2_(2, 154) = 3.08, p < .05. As the pattern of priming differed across words and nonwords, a series of pairwise comparisons were conducted for words and nonwords separately to elucidate where the differences in priming occurred.

For words, significant identity and scrambled priming effects were found relative to the unrelated prime condition, *F*_1_(1, 23) = 75.51, *p* < .001, *F*_2_(1, 77) = 58.62, *p* < .001 and *F*_1_(1, 23) = 12.68, *p* < .01, *F*_2_(1, 77) = 9.88, *p* < .01, respectively. The identity prime condition also differed significantly from the scrambled prime condition, *F*_1_(1, 23) = 15.73, *p* < .001, *F*_2_(1, 77) = 19.10, *p* < .001. Thus, responses times for words preceded by identity primes were 38 ms faster than scrambled primes, which in turn were 30 ms faster than unrelated primes.

Nonword RTs for the identity prime condition differed significantly from both the scrambled prime and unrelated prime conditions, *F*_1_(1, 23) = 6.16, *p* < .05, *F*_2_(1, 77) = 7.21, *p* < .01 and *F*_1_(1, 23) = 7.71, *p* < .05, *F*_2_(1, 77) = 13.35, *p* < .001 respectively. Importantly, the scrambled prime condition did not differ significantly from the unrelated prime condition, *F*s < 1. Thus, nonword targets preceded by an identity prime were responded to 30 ms faster than both scrambled and unrelated primes.

The analysis of error rates in the "same" trials revealed a significant effect of String Type, *F*_1_(1, 23) = 44.02, *p* < .001, *F*_2_(1, 153) = 4.54, *p* < .05 and a marginal effect of Prime Type by-participant, *F*_1_(2, 46) = 2.93, *p* = .06, and a significant effect of Prime Type by-item, *F*_2_(1, 153) = 4.57, p < .01. There was no interaction between String Type and Prime Type, *F*s < 1. Pairwise comparisons for data collapsed across String Type revealed that identity primes differed from scrambled and unrelated primes, *F*_1_(1, 23) = 4.55, *p* < .05, *F*_2_(1, 155) = 3.91, *p* < .05 and *F*_1_(1, 23) = 4.14, *p* < .05, *F*_2_(1, 155) = 8.11, *p* < .01, respectively. There was no difference between scrambled and unrelated primes, *F*s < 1.

#### "Different" trials

The analysis of RTs in "different" trials showed that the effect of String Type was not significant by-participant, *F*_1_(1, 23) = 1.80, *p* = .19, but was significant by-item, *F*_2_(1, 155) = 5.85, *p* < .05. There was no effect of Prime Type, *F*_1_(2, 22) = 2.56, *p* = .09, *F*_2_(2, 154) = 1.48, *p* = .23, and no interaction, *F*s < 1.

Analysis of error rates revealed a similar pattern; a significant effect for String Type by-participant, *F*_1_(1, 23) = 28.37, *p* < .001, but not by-item, *F*_2_(1, 154) = 2.44, *p* =.12, no effect of Prime Type, *F*_1_(2, 22) = 1.72, *p* = .19, *F*_2_(2, 154) = 2.33, *p* = .10, and no interaction, *F*_1_(2, 46) = 1.13, *p* = .33, F_2_ < 1.

### Discussion

Results from this experiment again revealed a lexicality effect. However, in contrast to Experiments 1a-d, a significant interaction emerged between string type and prime type when reference stimuli were presented in the auditory domain, demonstrating a different pattern of priming across words and nonwords. Specifically, scrambled primes produced a facilitation effect for word targets but not for nonword targets. Thus, the lack of scrambled priming effects for nonwords differs from the results of Experiments 1a-d, where scrambled priming effects were found consistently for both nonwords and words.

These results are consistent with the hypothesis that the matching process occurs at multiple levels for words but only at the sublexical level for nonwords [20]. One possibility is that when reference stimuli are presented in the auditory modality the target has to be converted to phonology to perform the same-different task. In this instance, scrambled primes facilitate processing of words at the lexical level through activation of shared orthographic representations such as open-bigrams. This does not occur for nonword targets, as they do not have lexical representations.

An alternative possibility is that auditory reference stimuli are converted to orthography and that matching occurs at the orthographic level (we thank a reviewer for this suggestion). In this case, the interaction found between string type and prime type could have arisen from ambiguity in the spelling of the spoken nonword reference stimuli. Thus, ambiguity of spelling could impact on scrambled priming for nonwords as there could be multiple spellings. No ambiguity would arise for matching auditory word reference stimuli to visual word targets, as the target words used in the experiment had only one possible spelling.

To test this hypothesis a control experiment (Experiment 2b) was conducted without nonword stimuli, as it is virtually impossible to create nonwords with unambiguous spellings. Instead, to manipulate ambiguity of spelling across the word stimuli, the experiment included two types of reference word stimuli: heterographic homophones, i.e. words that are spelt differently but have the same pronunciation (e.g., THEIR and THERE) and nonhomophonic control words.

Heterographic homophones provide an interesting way to test if ambiguity in spelling affects the pattern of priming, as heterographic homophone pairs generally consist of one spelling that is higher in frequency than the other (e.g., BOARD has a frequency of 64 versus BORED with a frequency of 20 per million). Several experimental paradigms have shown that this difference in written frequencies results in dominance for the higher frequency spelling [37]. This effect of spelling dominancy is extremely robust and is not influenced by recency effects and spelling regularity [38]. Furthermore, when required to spell an auditory-presented heterographic homophone the spelling with the highest frequency is given in almost all cases [37].

Thus, when presented with auditory reference stimuli that are heterographic homophones we predict that the dominant, higher frequency, spelling will be more likely to be activated than the lower frequency spelling. As a consequence, responses should be faster to targets with dominant compared to non-dominant spellings. Furthermore, if the auditory reference stimulus is converted to an orthographic code a different pattern of priming would be expected for dominant compared to non-dominant spellings. Scrambled priming effects should be observed with dominant spellings of the homophones, whereas no scrambled priming is expected for non-dominant spellings (where the auditory reference will create spelling ambiguity). Alternatively, if the target is converted to phonology to match to the auditory-presented reference, the pattern of priming should be similar across dominant and non-dominant spellings. Thus, if the match occurs at the phonological level there should be no interaction between homophone dominance and prime type.

## Experiment 2b

### Methods

#### Ethics statement

This experiment was approved by the School of Psychology Ethics Committee, University of Nottingham. Written informed consent was obtained from all participants prior to participation.

#### Participants

Twenty-four undergraduate and postgraduate students (16 females and 8 males, mean age 22.4 years) participated in this experiment. All were native English speakers with normal or normal-to-corrected vision.

#### Stimuli and design

Seventy-eight heterographic homophone word pairs (156 words) were selected from a list of 207 presented in Gorfein and Weingartner [37]. Homophone pairs were selected that matched in length (M = 4.7), but differed in spelling dominance as measured by word frequency (196 vs. 16 occurrences per million according to the SUBTLEX-US database [39]). Two lists of homophone pairs were created, matched for frequency, for "same" and "different" trials (all *t* < 1). A set of 156 control words (78 words for the "same" and "different" trials) were selected from the SUBTLEX-US database to match in length and written frequency to each of the 156 homophones (all *t* < 1). A further set of 156 words was selected as reference stimuli for use in the "different" trials. The three priming conditions, identity, scrambled and unrelated, were created using the same method as described in Experiment 1a. Homophones were fully counterbalanced across same-different trials and priming condition. Thus, in total six lists were created. Each participant was randomly assigned to one of the six lists. All auditory reference stimuli were recorded using the same method described in Experiment 2a.

#### Procedure

The procedure for this experiment was the same as for Experiment 2a.

### Results

Trials with latencies over 1400 ms or below 250 ms were removed from the analyses, accounting for 0.4% of the total data. The overall error rate was 5.2%.

To investigate the effect of homophone dominancy and the pattern of priming a 2x3 repeated ANOVA was conducted with Homophone Dominancy (dominant vs. non-dominant) and Prime Type (identity, scrambled, vs. unrelated) as independent variables. Mean RTs and error rates are presented in Table 4.

**Table 4:** Mean response times in milliseconds, percentage errors, and standard error (SE) of the means of Experiment 2b.

				Response Times (SE)		% Error (SE)	
Trials	Prime Type	Spelling Dominance	Prime - Target Pair Examples	Homophones	Controls	Homophones	Controls
"Same"	Identity	High	birth - BIRTH ^a^	492 (23)	464 (17)	3.4 (1.7)	5.4 (2.4)
		Low	berth - BERTH ^b^	565 (30)	448 (14)	8.3 (2.3)	3.8 (1.6)
	Scrambled	High	rbhit - BIRTH ^a^	511 (17)	496 (17)	3.9 (1.4)	5.2 (1.5)
		Low	rbhet - BERTH ^b^	597 (23)	494 (14)	7.7 (2.6)	0.6 (0.6)
	Unrelated	High	calls - BIRTH ^a^	528 (16)	517 (17)	7.3 (2.2)	10.3 (1.8)
		Low	calls - BERTH ^b^	633 (21)	542 (15)	12.3 (2.6)	3.7 (1.6)
"Different"	Identity	High	exit - EXIT ^c^	543 (22)	530 (21)	12.3 (2.6)	5.6 (1.7)
		Low	exit - EXIT ^d^	530 (18)	519 (19)	4.3 (2.0)	5.9 (1.9)
	Scrambled	High	xtei - EXIT ^c^	552 (23)	524 (25)	11.8 (2.1)	4.3 (1.6)
		Low	xtei - EXIT ^d^	537 (21)	522 (16)	3.7 (1.6)	5.7 (2.5)
	Unrelated	High	such - EXIT ^c^	542 (17)	533 (19)	11.4 (2.3)	5.8 (2.5)
		Low	such - EXIT ^d^	538 (21)	541 (18)	3.6 (1.6)	3.7 (1.3)

a Reference: birth

b Reference: berth

c Reference: warn

d Reference: worn

#### "Same" trials

The latency analysis revealed a significant effect of Homophone Dominancy, *F*_1_(1,23) = 45.30, *p* < .001, *F*_2_(1,78) = 26.22, *p* < .001, with responses to dominant homophone spellings 88 ms faster than non-dominant spellings. A main effect of Prime Type was found, *F*_1_(2,46) = 10.59, *p* < .001, *F*_2_(1,78) = 14.43, *p* < .001. Importantly, no significant interaction was obtained, *F*_1_(2,46) = 1.76, *p* =.18, *F*_2_(1,155) = 2.78, *p* = .07.

To investigate the main effect of Prime Type, RTs were collapsed across Homophone Dominancy and planned comparisons were conducted. These revealed that identity and unrelated primes differed significantly, *F*(1,23) = 8.64, *p* < .05, *F*_2_(1,78) = 22.17, *p* < .001. Scrambled primes were faster than unrelated primes but this difference just failed to reach significance (2-tailed), *F*(1,23) = 4, *p* = .06, *F*_2_(1,78) = 3.72, *p* = .06. Identity primes also differed significantly from scrambled primes, *F*(1,21) = 4.75, *p* < .05, *F*_2_(1,78) = 10.52, *p* < .01. Thus, identity primes were responded to faster (24 ms) than scrambled primes, and scrambled primes were faster (27 ms) than unrelated primes.

Error rates revealed a significant effect of Homophone Dominancy, *F*_1_(1,23) = 4.19, *p* = .05, *F*_2_(1,77) = 9.14, *p* < .01, with responses to dominant spellings 4.8% more accurate than non-dominant spellings. A significant effect of Prime Type was found by-participant, *F*_1_(2,46) = 3.60, *p* < .05, but not by-item, *F*_2_(1,154) = 2.25, *p* =.11. Importantly, no significant interaction was found, *F*s < 1.

#### "Different" trials

Responses latencies and error rates revealed no significant main effects for Homophone Dominancy, Prime Type, and no interactions, all *F*s < 1.

#### Homophones versus controls

To explore the general effect of homophones compared to control words in relation to priming condition, 2x3 repeated ANOVAs were conducted with Word Type (homophones vs. control words) and Prime Type (identify, scrambled, vs. unrelated) for the "same" and "different" trials separately.

As expected, the latency analysis of the "same" trials revealed a significant main effect of Word Type, *F*_1_(1,23) = 40.96, *p* < .001, *F*_2_(1,155) = 80.68, *p* < .001, with responses to homophones (where there is spelling ambiguity) 60 ms slower than control words. Again a main effect of Prime Type was found, *F*_1_(2,46) = 19.85, *p* < .001, *F*_2_(1,155) = 43.81, *p* < .001, and the interaction was not significant, *F*s *< 1*.

To investigate the main effect of Prime Type for the "same" trials, RTs were collapsed across Word Type and planned comparisons were conducted. These revealed that both the identity and scrambled primes differed significantly from the unrelated primes, *F*(1,23) = 27.56, *p* < .001, *F*_2_(1,155) = 109.5, *p* < .001, and *F*(1,23) = 13.18, *p* < .01, *F*_2_(1,155) = 10.85, *p* < .01, respectively. Identity primes also differed significantly from scrambled primes, *F*(1,21) = 12.35, *p* < .01, *F*_2_(1,155) =29.13, *p* < .001. As before, identity primes were responded to faster (32 ms) than scrambled primes, and scrambled primes were faster (31 ms) than unrelated primes.

Error rates of the "same" trials revealed a marginal effect of Word Type by-item, F_1_< 1, *F*_2_(1,77) = 3.48, *p* = .06, and a significant main effect of Prime Type, *F*_1_(1,23) = 5.12, *p* < .01, *F*_2_(1,77) = 4.99, *p* < .01. No significant interaction was found, *F*s < 1. When collapsed across Word Type, identity primes were significant more accurate than unrelated primes (3.2%), *F*_1_(1,23) = 4.78, *p* < .05, *F*_2_(1,77) = 4.44, *p* < .05. Scrambled primes were significantly more accurate than unrelated primes (4.1%), *F*_1_(1,23) = 9.30, *p* < .05, *F*_2_(1,77) = 12.96, *p* < .001. The difference in error rates between identity and scrambled primes (.8%) was not significant, *F*s < 1.

Both response latencies and error rates of the "different" trials revealed no significant main effects for Word Type, (RTs: *F*_1_(1,23) = 3.67, p = .07, F_2_ < 1), Prime Type, and no interactions, all *F*s < 1.

### Discussion

This experiment was conducted to test the prediction that the lack of scrambled priming in nonwords observed in Experiment 2a, when reference stimuli were presented in the auditory domain, arose through spelling ambiguity. Here, we manipulated spelling ambiguity explicitly through using heterographic homophones with dominant and non-dominant spellings.

As expected, responses were faster to targets with dominant than non-dominant spellings. Importantly, we found no interaction between homophone dominancy and prime type. Thus, spelling dominancy did not modulate scrambled priming effects. This suggests that when the reference stimulus is presented in the auditory domain the target is converted to a phonological code and the match occurs at the phonological rather than the orthographic level. Furthermore, responses to homophones were slower than to control words and a similar pattern of priming was found.

## General Discussion

This study investigated the origin of the lexicality effect shown consistently in the same-different task. The overall pattern of results in Experiments 1a-d showed a consistent processing advantage for words over nonwords (magnitude of the lexicality effects 1a: 25 ms, 1b: 29 ms, 1c 27 ms, 1d: 24 ms). Critically, the pattern of masked priming effects was the same for words and nonwords. Both the lexicality effects and patterns of priming found in Experiments 1a-d were independent of the duration of the reference stimuli and the predictability of the target string type (both between and within trials). However, Experiment 2a in which reference stimuli were presented in the auditory domain revealed a different pattern of priming. In particular, a significant scrambled priming effect was observed for *words only*. Furthermore, ambiguity in nonword spelling could not account for the scrambled priming effect because when the task was conducted with heterographic homophones (Experiment 2b) the scrambled priming effect remained.

The current study provides compelling evidence that the advantage for processing words is due to the activation of whole word lexical representations [20,21]. This lexicality effect supports the theory that matching in the same-different task can occur at several different levels [20], with nonwords matching at the sublexical level and words at the lexical and sublexical levels.

The lexicality effect, which is shown clearly across all of our experiments, is problematic for the Bayesian Reader model [19] as it predicts that the representations and evidence, both perceptual and priors, used to make the decision in this task are nonlexical in nature. Thus, the Bayesian Reader model predicts that both matching and priming occur at the same level for words and nonwords. The presence of a lexicality effect in our experiments indicates that different representations are used across words and nonwords.

A key result of this study is that scrambled priming effects occurred for words *only* when the reference stimuli were presented in the auditory domain (Experiment 2a). Even when spelling ambiguity was manipulated across words by using heterographic homophones, scrambled priming effects were shown (Experiment 2b). Our results are consistent with the assumption that when the reference stimulus is presented in the auditory modality the matching process occurs at the phonological level, therefore the target has to be converted into a phonological code. For words this could occur at the lexical or sublexical level but for nonwords this is only possible sublexically. When letter order is preserved, as in identity primes, conversion of the target to phonology is facilitated for both words and nonwords, but when letter order is disrupted, as in scrambled primes, conversion of the target to phonology is not facilitated at the sublexical level. However, as we suggested, scrambled primes could still potentially facilitate the processing of word targets at the lexical level through the activation of shared sublexical orthographic representation (e.g., open-bigrams) between the prime and target. For example, although scrambled primes do not contain contiguous positional information, they can still contain non-contiguous positional information (e.g., SOUTH scrambled becomes USHOT, in which the open-bigrams SO, SH, UH, and UT are preserved) and thus they can activate the lexical representations. In contrast, scrambled priming effects cannot occur for nonword targets because they do not have lexical representations.

If scrambled primes contain just letter identity information, as argued by Kinoshita and Norris [19], priming should occur for both words and nonwords. Importantly, the results of Experiment 2a revealed that scrambled priming effects occurred *only* for words, confirming that scrambled primes are able to activate lexical representations in the same-different task. This supports the hypothesis that lexical effects operate in the same-different task.

Several models of visual word recognition include open-bigrams to encode position (e.g., [4,5,9,40]). These models contain two different types of open-bigrams, contiguous, with adjacent letters (e.g., FA, AI, IT, TH, for FAITH) and non-contiguous with non-adjacent letters in the correct order but with one or more intervening letters (e.g., FI, FT, FH, AT, AH, IH). Depending upon the particular model different constraints to the type of open-bigrams and the distance separating the two component letters have been proposed. For example, Grainger and van Heuven [4] used both contiguous and non-contiguous open-bigrams, with a distance of no more than two intervening letters separating component letters. Close examination of the scrambled primes used in the current experiments revealed that they shared four out of nine possible open-bigrams with the target, one contiguous and three non-contiguous. Thus, the scrambled primes not only matched the targets in terms of their letter identity but they also contained some relative positional information. Thus, it is possible that the effects of priming in the scrambled condition are due to the number of shared open-bigrams with the target.

**Figure 2:**
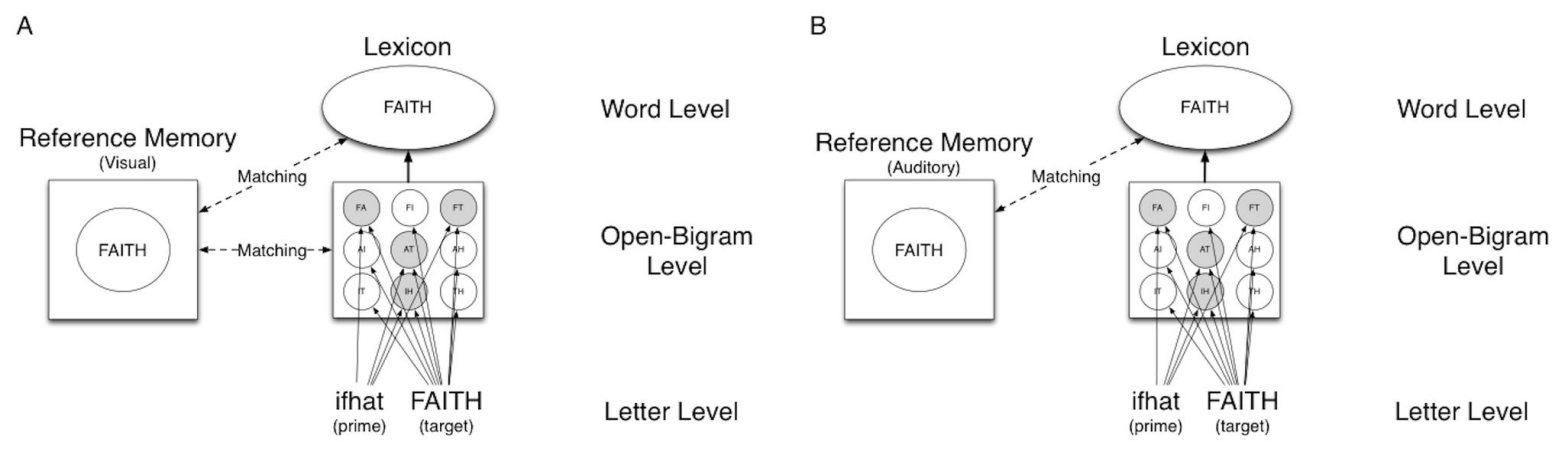
Model of the masked priming same-different task with visual (A) and auditory (B) reference stimuli and a scrambled prime. Gray-filled circles at the Open-Bigram Level indicate shared open-bigrams between prime and target.

Figure 2 illustrates a proposed model of the same-different task based on Chambers and Forster [20] that involves open-bigrams as in the model of Grainger and van Heuven [4]. This model shows how matching in the masked same-different task occurs when the reference stimulus is presented in either the visual or auditory domain. When the reference is presented in the visual domain, nonword matching occurs at the open-bigram level, whereas matching for words occurs at either the open-bigram or word level. Thus, this model predicts that scrambled priming effects occur for both words and nonwords when the reference is presented visually. The model is also compatible with the key finding of Experiment 2a in which no scrambled priming for nonwords was found when the reference was presented in the auditory domain so matching cannot occur at the orthographic level. The matching process for nonwords must therefore occur through conversion of the visual target to phonology. This is supported by longer reaction times for Experiment 2a than Experiment 1a.

Although the results from Experiments 1a-d showed no significant interaction between string type and prime type, further exploration of the effects of prime type on identity and scrambled priming for words and nonwords independently were conducted (we thank a reviewer for this suggestion). Table 5 shows no significant word advantage in the identity priming condition across Experiment 1 confirming our earlier analyses. Likewise, no significant word advantage was found across the scrambled priming condition in Experiment 1 when the reference duration was relatively long (i.e. ≥ 1000 ms). However, in Experiment 1c with the short reference duration (500 ms) there was no significant scrambled priming effect for the nonwords, whereas for the words there was a significant and moderate to large scrambled priming effect (Cohen’s d = .70). In addition, the difference in magnitude of the scrambled priming effect across words and nonwords was significant (*p* < .05), whereas for identity priming there was no word advantage (*t*(32) < 1). Seemingly the duration of the reference stimulus influences the extent of scrambled priming for nonwords. This may be accounted for by lexical processing in that the short duration of the reference might be sufficient to activate lexical representations that are similar to the reference (e.g. orthographic neighbors) which feedback to prelexical processes. Further investigations are required to determine the precise mechanisms that give rise to this effect of reference duration. Interestingly, Table 5 also shows that the significant interaction found in Experiment 2a, when the reference stimulus was presented in the auditory domain, arises from a significant word advantage in both the identity and scrambled priming conditions. This adds further confirmation that the visual target is converted to a phonological code.

**Table 5:** Significance, magnitude, and effect sizes (Cohen’s *d*) of identity and scrambled priming (in milliseconds) for Experiments 1a-d and 2a.

			Identity Priming			Scrambled Priming		
Experiment	Reference Modality	Reference Duration	Words (*d*)	Nonwords (*d*)	Word Advantage	Words (*d*)	Nonwords (*d*)	Word Advantage	
1a	Visual	1000 ms	46** (1.8)	45** (2.1)	*t*(23) < 1	16** (0.58)	22** (0.84)	*t*(23) < 1	
1b	Visual	2000 ms	47** (0.92)	36** (0.85)	*t*(40) = 1.15, p = .26	17* (0.4)	10 ns (0.26)	*t*(40) < 1	
1c	Visual	500 ms	31** (0.55)	23* (0.47)	*t*(32) < 1	23** (0.70)	6 ns (0.16)	*t*(32) = 2.70, *p* < .05	
1d	Visual	1000 ms	61** (1.94)	50** (1.35)	*t*(23) = 1.25, *p* = .22	35** (1.23)	24** (0.69)	*t*(23) = 1.40, *p* = .17	
2a	Auditory	1000 ms	68** (1.77)	30** (0.6)	*t*(23) = 2.29, *p* < .05	34** (0.73)	4 ns (0.13)	*t*(23) = 2.66, *p* < .05	

Note. *p < .05, **p < .01; Prime Type x String Type interaction was only significant in Experiment 2a.

To summarize, the proposed model presented in Figure 2 can account for the scrambled priming effect that was found for words independent of the modality of the reference stimuli. Furthermore, it can account for the finding that the scrambled priming effect for nonwords depends critically on the reference modality because a scrambled priming effect was found with a visual reference (Experiment 1) but not with auditory reference stimuli (Experiment 2) as revealed by a between-experiment analysis of Experiments 1a and 2a with the scrambled and unrelated conditions in which no interaction was found for words, *F*_1_(1, 23) = 2.1, *p* = .15, *F*_2_(1, 154) = 1.40, *p* = .24, whereas for nonwords a significant interaction was found by-participant, *F*_1_(1, 23) = 4.1, *p* < .05, *F*_2_(1, 154) = 1.56, *p* = .21. In addition, match values calculated using Colin Davis’ Match Calculator (http://www.pc.rhul.ac.uk/staff/c.davis/Utilities/MatchCalc/) showed that Grainger and van Heuven’s model [4] produces higher match scores (.44), for the overlap between the scrambled primes and their targets, than other models of orthographic processing which encode letter order using relative positional information, such as SOLAR [1,2], SERIOL [41], and Overlap model [3] (match scores: SERIOL = .28, SOLAR = .41, Overlap = .35). Alternative models such as the hybrid model of visual word recognition [42] that suggest a whole-word channel based on different spatial frequencies may also account for the overall pattern of results reported here. At present this model is not implemented so match scores are unavailable. We are currently comparing an implemented version of the proposed model in Figure 2 to other implemented models of visual word recognition. In addition, holistic models such as Marmurek [21] and Allen et al. [42] would predict case effects between the reference, prime and target, whereas models utilizing abstract letter units (e.g., [4]) would not. Further experimentation manipulating case (e.g. "flair" as the reference, "flair" as the prime, and "FLAIR" as the target compared to "flAIR" as the reference, "flair" as prime, and "FLair" as the target) is required to distinguish these accounts.

In conclusion, the results of the experiments reported in this study demonstrate that the lexicality effect shown in the masked priming same-different task arises from the activation of different sized representations for words and nonwords. Specifically, words activate lexical and sublexical representations, whereas nonwords only activate sublexical representations. Thus, our data provide evidence for lexical influences in the masked priming same-different task and constrain the interpretation of priming effects found in previous studies using this task. Furthermore our pattern of findings suggests that lexical activation may well be an obligatory consequence of experimental tasks that involve the presentation of real word stimuli. Indeed it remains to be seen whether or not it is possible to develop tasks, and/or methods of data analysis, that enable the dissociation of lexicality effects from purely prelexical orthographic processes when employing words as stimuli. This is a key challenge for future research in this area but for the present it is sufficient to conclude that the use of real words should be avoided when attempting to isolate the mechanisms that mediate sublexical processing of letter strings.
